# A Grounded theory study of the intention of nurses to leave the profession**^1^**

**DOI:** 10.1590/1518-8345.1638.2894

**Published:** 2017-06-05

**Authors:** Leyla Alilu, Vahid Zamanzadeh, Leila Valizadeh, Hosein Habibzadeh, Mark Gillespie

**Affiliations:** 2 PhD, Assistant Professor, Department of Medical Surgical Nursing, Faculty of Nursing and Midwifery, Urmia University of Medical Sciences, Urmia, Iran.; 3 PhD, Professor, Department of Medical Surgical Nursing, Faculty of Nursing and Midwifery, Tabriz University of Medical Sciences, Tabriz, Iran.; 4 PhD, Associate Professor, Department of Pediatric Nursing, Faculty of Nursing and Midwifery, Tabriz University of Medical Sciences, Tabriz, Iran.; 5 MEd, Nursing Lecturer, School of Health Nursing and Midwifery, University of the West of Scotland, Paisley, Scotland.

**Keywords:** Grounded Theory, Personal Turnover, Nurses, Iran, Qualitative Research

## Abstract

**Objective:**

this study explores the process of the development of an intention to leave bedside nursing.

**Method:**

the process was studied from the perspective of 21 nurses using the grounded theory method. Data were collected using semi-structured interviews and the constant comparative method of Corbin and Strauss was used for data analysis.

**Results:**

according to the participants, the two main categories, "social image of nursing", and "culture and structure of the bedside", were the contextual factors that influence why nurses are leaving bedside care provision. Disappointment with a perceived lack of progress or improvement in the clinical experience formed primary psychosocial concerns for the participants. Competence and a process of self-control were steps taken by the participants. These, associated with interventional conditions produced the outcomes of the loss of professional commitment and desire to leave bedside nursing. "Failure to integrate personal expectations with organizational expectations: in search of escape" was the central category of the study that linked the categories together.

**Conclusion:**

the findings of this study provide useful information about the needs of nurses for overcoming the intention to leave bedside care. The identification of this process can help in recognizing emerging problems and providing solutions for them.

## Introduction

Turnover is a critical issue for the nursing workforce^1^, negatively affecting the health system regarding both disease prevention and the health and quality of the nursing services^2^. The shortage of nurses is a global problem^3^ and the turnover of nurses is very high in comparison to other healthcare professions^4^. The Council of Europe has estimated that the nursing workforce will face a shortfall of 590,000 people by the year 2020 and the intention to leave the nursing profession varies from 4% to 54% of nurses surveyed^5^. In addition, almost 72% of nurses have thoughts of leaving the nursing profession everyday^6^. In Iran, official figures on the shortage of nurses are not available, however, with a population of 75 million in 2013, it is considered that approximately 240,000 nurses are needed to service this size of population, whereas there are currently only 100,000 nurses. Even by doubling that number, the population would still have minimal access to health care^7^. In other studies with Iranian nurses the levels of intent to leave nursing are high^8^ and only one third of nurses (34%) reported satisfaction with their jobs^9^. Lack of job satisfaction is likely to have a direct impact on the intent to leave the career. Given the current shortage of nurses in the country, and considering the severe shortage of nurses expected in the future due to the retirement of existing nurses, population growth, and an aging population profile^10^, it is of extreme importance to assess the extent of this problem in Iran.

A shortage of nurses and high staff turnover are recognized as detrimental to patient care^11^, quality of care^12^, and healthcare costs^13^. Costs associated with staff turnover including recruitment, financial and training issues contribute towards organizational dysfunction^14^. 

To understand the decision making process through which individual nurses are influenced towards the consideration of leaving their profession requires application of a research methodology that is sensitive to individual decision making, set against the context of wider social interaction^15^. Such a depth of understanding cannot be achieved meaningfully through the use of questionnaires and closed questions. For this reason, the present study utilized a qualitative methodology in the form of grounded theory. 

It is recognized that nurses working within Iran may face unique influences on their decision making, regarding decisions to leave nursing, or may make decisions in different ways, indeed one study identified quite different results in comparison to nurses studied elsewhere. This study found that although the nurses in Iran were not entirely happy in their work, they did not want to leave it. The author of the study was testing a model to explain this decision making process and, as a consequence of the results obtained, suggested it as an additional dimension in the development model. He proposed that other researchers investigate this concept in Iran, carrying out studies to explore the role of factors such as the labor market and employment status in this field^16^. This paper discusses the reasons that lead to an intention to leave the nursing profession, it reviews the context of decision making with regard to leaving bedside care, and studies the conditions influencing this. It is hoped that by understanding these processes, the relevant authorities will be able to formulate a strategy to retain nurses within the clinical practice. The aim of this study was to explore the developmental process of the generation of a desire to leave bedside nursing. 

## Method

The nurses who participated in this study were selected from several teaching hospitals affiliated to Tabriz and Urmia Universities of Medical Sciences in Iran. The sample selection process was based on the following criteria: 1) having a high school diploma or higher, 2) having at least one year of work experience in the clinical nursing practice, and 3) acceptance of the invitation to participate in the study. A total of 21 nurses employed in different wards of a governmental teaching hospital met the inclusion criteria and agreed to participate in the study.

Selection of the participants was initiated using purposive sampling and after the eighth interview continued through theoretical sampling (In order to complete the categories and concepts created from previous interviews) until saturation was achieved.

 The study subjects took part in semi-structured interviews in which open-ended questions were used to investigate the process of the development of the intention to leave the profession in experienced nurses working in a clinical setting between May 2014 and July 2015, (over a period of 14 months). Researchers interviewed each participant individually for 40 to 60 minutes in the work place (n=16), and outside (n=5). The interview began with the general question "how would you describe your experience of the process of desire to stop being a clinical nurse?", and moved to more specific and detailed questions as the interview advanced. These included "What factors or circumstances make you feel like leaving bedside care?" "What strategies did you use to overcome any thoughts of leaving bedside care?" and "Give examples of your experiences".

The interviews were recorded with the participant's permission, and they were later transcribed. The raw data were coded Verbatim using MAXQDA10 (version 10 R 160410 by udo kuckartz, Berlin, Germany), prior to the analysis. Nineteen out of the twenty one participants in the study were female and the others male. Participant ages ranged from 24 to 45 years, with a mean age of 32 years and mean work experience of 6 years (1.5 to 18 years). Thirteen of the participants graduated with a bachelor's degree in nursing, while eight of them had a Master's qualification in nursing. Among the participants, 11 were married and 10 were single.

This was a qualitative study, based on the Grounded theory approach. The Grounded theory methodology^17^ is rooted in landscape interpretation and symbolic interaction, and it suggests that reality exists in the meaningful social actions of individuals, which are created through interpretational interactions. The constant comparative analysis of Corbin and Strauss was used to analyze the data. This process is composed of three distinct phases: open coding, axial coding and selective coding. Data collection and analysis in the research process occurred simultaneously^17^ and the emerging theory was proposed based on the data. Data analysis continued simultaneously after the first interview until saturation was reached. Researchers encrypted the copied text, and discussed coding refinement for each emerging theme. Classified codes were categorized, compared and interpreted within the context of the general transcripts. 

For reporting of the qualitative study findings, the trustworthiness of methods are widely considered applicable in place of the validity and reliability associated with quantitative research^18^. In the present study four supporting processes of trustworthiness were applied, namely conformability, dependability, credibility and transferability. Credibility was confirmed by selecting the appropriate data collection method for the interviews. The researchers interviewed participants for their views and experiences in their practice environment. Furthermore, member check was used to prolong the involvement of the researcher to increase the credibility of the data and, after encoding, the interview transcripts were returned to the participants to ensure the accuracy of the codes and the relevant interpretations. Dependability was established by detailed and descriptive data analysis and direct references to the professional experiences of the individual. Raw data were translated by a professional translator from Farsi (Persian) into English and back translated to preserve maximum accuracy of participant expressions within the context. The conformability and consistency of the analysis were maintained through research team meetings to discuss and dissect the preliminary findings. Thematic analysis and the coding process occurred through consensus, and to increase the transferability of the findings, a description of the context, selection and demographic data of the participants, data collection and the analysis process was presented so that the reader would be able to determine whether the results are transferable to other environments^19^_._

The study was approved by the Ethics Committee of Tabriz University of Medical Sciences (no. 5/4/3861), Iran. Prior to data collection, the researchers obtained written informed consent of the participants, with the guarantee of anonymity, privacy and confidentiality and assurance of the voluntary nature of their participation. Information on the study objectives and goals were explained in detail, and contact information of the principal investigator was provided to answer any questions of the participants.

## Results

Data were presented using the framework proposed by Corbin and Strauss for the development of categories and subcategories. The categories include: causal conditions, Phenomena (how did the participants experience intending to leave bedside care), the context in which it happened, the interventional terms that affected the coping strategies of the participants, action/reaction (coping strategies), and the consequences of choosing their coping strategies. Finally, the categories were connected together and the meaning of "mismatch of expectations: in search of escape" was theorized.

### Causal Conditions

Two subcategories were extracted from the category of causal conditions (contributing to the intent to leave bedside care), with them providing an explanation about how the phenomenon of considering leaving the clinic practice was experienced by the participants. Causal factors for this consisted of powerlessness and feelings of worthlessness.

### Powerlessness

Based on the experiences of the participants, it was found that clinical nurses lacked the necessary power to value nursing and they did not feel supported by those in authority. Furthermore, the experiences showed that the present physician-centered strategies in the hospitals and some other factors contributed to the nurses deciding to leave bedside care. The experience of being a subordinate, as proposed by some participants, was another relevant factor in this, with the following comments from the participants: *A Doctor came and asked why I did not inject the patient with insulin? I said when his blood glucose is 84, why should I inject insulin? ... But the doctor said you should, because it is an order. I answered, well; if I had and his blood sugar had dropped to 30 ... would you not have queried me?* (p14); *so why do they despise nurses? The nurse is responsible for the wrong decisions of the doctor ... in the services any workers' fault is wrongly related to the nurse! .... I do not like this work and I really want to quit this job*... (p15); *the nurse must request permission from the physician for anything*... (p9); *I've reached the point where I can no longer stay...The physicians look at the nurse as just a subordinate*. (p13)

### Feelings of worthlessness 

Feelings of worthlessness are what make people believe that their efforts do not matter; hence they try to escape from the situation. In this study, this feeling was described based on the experiences of the participants with characteristics of 'humiliation' and 'frustration'. Referring to this, the participants said: *I feel humiliated... I work in a low level and worthless profession ... As a result, I always feel frustrated. And sometimes I regret choosing this profession.* (p1); *I was not valued even as a nursing expert. That is, they looked at me negatively... when I made a suggestion, they did not pay any attention to me.* (p11)

### Phenomena

The causal conditions led to disappointment regarding opportunities to progress and perceived lack of improvement in the clinical setting by the participants, with them despairing about progress and improvement in the clinical practice

The nurses said they had no power at the bedside, and they felt there was no improvement or progress in the healthcare system. Their clinical skills were ignored and neglected by their managers; meanwhile any minor flaws they exhibited were magnified and blown out of proportion. Due to the fact that bedside care did not fully satisfy their needs, they were seeking other opportunities to satisfy their needs related to ways to progress in the system. Therefore, their main concern was the lack of opportunity for professional development. *I think it's terrible that I graduated as a nurse, and I should work as a nurse for the rest of my life. It doesn't matter in which hospital or sector you are working, your work and status is the same all the time everywhere*. (p9); *There is nothing to encourage you, but there is always something to discourage you.* (p19); *I had a patient in a severe condition; I worked hard till morning... they did not appreciate me. But in the morning because the fluid in an IV was bloody, the manager's behavior was not correct and they said, 'Why did not change the fluid ....* (p2); *No matter what you do and even if you do your work in the best possible way, nobody is satisfied, and certainly a fault will be found with your work.* (P3); *Now, in our hospital, Promotion is not based on the ability of individuals, It is based on work experience and the level of flattery given to the Hospital manager or knowing someone in the Nursing hierarchy. When I think about it, I feel that I'm buried in a clinical setting; I am looking for a way to jump out.* (p11)

### Context

The contexts in which intent to leave bedside care emerged were classified into the following two categories: "Social image of nursing", "Culture and structure of bedside care"

### Social image of nursing

Most people do not understand the needs of the nursing profession or the expectations of the nursing role. The same can be said of the skills required of the nursing profession. Unfortunately, the attitude towards the nursing profession in Iran is not positive. A lack of understanding by patients and their families of the nursing profession is another factor affecting the nurses. The participant gave the following comments on this: *A patient's family member came in front of the station and said the nurse should clean her baby and his bed.... it is the duty of the nurse ... That is why you are paid. It is your duty... the family members don't know what the nurse's duties are*... (p16); *they disrespect us and they were saying "what kind of woman are you who are out of your house at night". Some people even associate nursing with lower classes in society. For example, they told "you are very needful to work here*". (p20)

### Culture and structure of the bedside care

Based on the experiences of the participants, there was a significant availability of work within nursing and workforce recruitment was prominent. The nursing work environment was, however, not ideal in terms of the ratio of patients to nurses, the high levels of violence against nurses, variations in shift patterns, high numbers of shifts, inadequate contracts, deficiencies in comparative salaries and working experiences in general. Nurses were forced to work in hostile environments with high expectations regarding the workload and the level of responsibility undertaken as a graduate nurse. In some shifts they had to fulfill the entire care needs with the help of only one auxiliary nurse. The number of nurses was low and levels of necessary patient care high. Some shifts had heavy workloads and the nurses could be extremely busy. The participants did not have much fun nursing and felt that activities outside of bedside care would be more rewarding than providing patients with good quality care. Recalling their night shifts revealed that at times the pressure felt was excessive. Contributing to this were the number of shifts worked, the wearing nature of double shifts and the demands of night shifts, which the nurses said led to tiredness. Feelings of guilt were generated as a result of feeling they had failed to provide sufficient care. The participants also felt that the hospital managers did not really understand how physically demanding nursing work is, and therefore had unrealistic expectations regarding the provision of patient care. 

All of the nurses questioned were dissatisfied with their wages. They believed the wages of nurses are too low in relation to the responsibility associated with the position. The participants said: *On the one hand, the workload is high, there is much pressure, the head nurse bothers others, and it bothered me so...* (p4); *our shift is very intensive and there is no understanding in the ward; the families of patients do not understand... the supervisors, too. All the people say go do your task. In the past there was a head nurse, we had a terrible situation. We could not sit for a moment, because if she knew that someone was sitting she would report them immediately, so I finished my work and then I would sit...* (p13)

### Interventional Conditions 

The coping strategies of the participants were also affected by the interventional conditions (conditions prohibiting nurses from leaving bedside care) "having a role in society" and "having a job and income".

### Having a role in society

Some participants stated that they tolerated the poor clinical conditions because it gave them a role in the family and society. This is because when they work, they are considered part of the community and thereby have a role to fulfill. Participant 6 said: *I work here to avoid being penniless and unemployed...I am therefore contributing to society. For example, in terms of financial help for my husband, sometimes I feel I have a significant role to play.*

### Employment and income

The majority of the participants believed that having a permanent job was a good thing as it allowed them control in their lives, however, they were also sure that they did not want to spend all of their time working. The most significant contributor to this was recovery from night shifts and consequential daytime sleep. The participants continued in bedside care not due to enthusiasm and interest but because leaving bedside care would be stressful. *I am not satisfied with my work, but for vocational security and financial need, because of use of my information in my work, I have to endure and continue*. (p7); *really working at the bedside has no value. Though, I would not work if I did not have financial need.* (p12)

### The action/reaction (strategies to overcome the intention to leave bedside care)

Coping strategies were affected by casual conditions (powerlessness and feelings of worthlessness) and the phenomenon (disappointed about promotions in clinical care). Nurses also developed their coping strategies as a result of the context in which the desire to leave bedside care occurred and the conditions surrounding this. The two major coping strategies used by them were 1) competence and 2) self-control. The participants frequently used both strategies.

### Competence

Competence includes developing features of professional capabilities, supporting research and striving to maintain a work-life balance. *As much as I can, I do research work. Because I know, we must promote our profession* (p4*); in a workshop that many doctors and health department members attended, I tried to work very well because I wanted to promote nursing* (p17); *I work in nursing wholeheartedly and that's not because of pressure from the head nurse* (p21). 

### Self-control

Self-control is another progressive process, where data from the statements of the participants included self-control shaped by the conditions generating concern. Describing characteristics of this class include "avoiding," "tolerance" and "routine-centered performance". *On one occasion I demanded that they change my ward*. (p10*); I'd rather not talk too much in the ward and be quiet. When a patient comes and I sense she is inconvenienced, I try to give convincing answers to her.... when I sense that she is not convinced I'd rather not discuss it any further and leave the room because I become more nervous.* (p8)

### Outcomes 

The participants used active and passive strategies to overcome perceived social and psychological problems, leading to subsequent consequences. These consequences were located in a spectrum ranging from being forced to leave bedside care through to persevering with it.

### Loss of effective professional commitment

The participants continue providing bedside care, however, not because of enthusiasm and interest, but because leaving before finding alternative employment would be stressful. At this stage participants had generally decided on a temporary stay while they continued to look for a position that would enable them to leave bedside care. The participants stated: *I only stay because of desperation* (p3); *it was here that 4 patients were intubated in our ward and we had been working with two nurses and 19 patients.... that is, there is nothing for me for all the services I offer, nothing financially or spiritually, I have lost interest in caring for the patients. I was affable. Now I have lost my interest, my program is: coming to the ward and I do some routines then go ... that feeling of interest is gone.* (p2)

### Leaving bedside care

To leave bedside care temporarily or permanently, and even thinking about it, may be the last resort for the participants in the study (lack of hope for the development and promotion of clinical work). *I found a morning shift job in the health institution, I want to go there. I do not want to stay in the bedside care.* (p5); *I am educated and have a Baby, and I applied to do a PhD. Just to escape from beside nursing.* (p11); *I resigned because I was tired*. (p18)

Corbin and Strauss stated that the central theme in a study is abstract, it will frequently appear in the data and almost all of the participants will relate to the concept. The derived central class in response to the question of how nurses deal with the experience of intending to leave bedside care is a "Mismatch of expectations: in search of escape". This theme made ​​the participants feel like leaving bedside care and, if allowed to continue and develop, triggered consequential coping strategies; and influenced the consequences of the coping strategies selected.

When they join an organization, nurses have specific values ​​and expectations regarding the development of personal knowledge, participation in decision-making, understanding how to upgrade flexible work schedules and so on. The participants felt that nursing did not fulfill their mental challenges after many years of working in the profession and that they did not have the opportunity to progress. They sought development opportunities, but had been unable to follow them in the practice. In fact, it can be said that based on the content of the participants statements, there was a conflict between the employing organizations and their staff and it was understood that the practitioners need to defer to the outcomes of the organization. If this is not anticipated it can ultimately lead to detriment for the organization through the loss of effective professional commitment and a motivation to leave bedside care.

## Discussion

By comparing these findings with other studies, it was found that these factors are identified in a variety of contexts. However, the intensity is different and this should be noted for the preservation and retention of nurses. 

The nurses who participated in the current study reported powerlessness and feelings of worthlessness in the clinical practice and complained about the lack of a proper view of the nursing discipline in the community, which impacts negatively on nurses. This finding is consistent with results from other studies. In another study performed in Iran, 70.3% of the nurses were not satisfied with the social status of the nursing profession in their community. Furthermore, 80.7% of the nurses identified that, due to the difficulties related to the profession, such as low pay, irregular working hours etc., they would not be satisfied if their children chose the nursing profession as a career^20^. If the relationship between doctors and nurses is not based on participation, equality, trust and respect and doctors look upon nurses as worthless and incompetent people, nurses will feel uncomfortable in their work and will not progress due to feelings of inferiority^21^. This shows that nursing professionals need to be valued and acknowledged for their work^22^.

According to the findings of this study, the culture and practice of bedside care is the main factor causing participants to leave this setting. A literature review showed that excessive workload, lack of facilities and support services, manpower shortages and insufficient ratios of nurses to patients were considered to contribute to the generation of the intention to leave nursing^23^_._

Constant comparison analysis of the data indicated that the main psychosocial problem, that is the main concern identified by the nurses participating in this study, was the lack of opportunities for advancement and promotion. Studies have shown that there is a relationship between individual performance and turnover. The imbalance between personal effort and the reward gained is directly related to the intention of nurses to leave^24^. This is what the participants referred to as 'weakness' in this study. 

The study participants faced limited opportunities for development and used strategies to overcome this, following the specified processes including the two steps of competence and self-control. The results of this study present differences and similarities with some national and international studies. An interesting finding of this study, contrary to similar research^25^, is that the coping strategies applied to overcome any intent to leave bedside care were more evident in person-oriented actions, while the role of the management and organization was weak. Another difference between this study and other studies^26^ ^-^ ^27^ is that the nurses here did not experience fatigue from the exposure to problems, even though they showed some degree of wanting to leave the bedside. The results of the investigation^28^ showed that empowerment is a predictor of job satisfaction and a factor supporting the retention of employees in the workplace and in determining whether they will avoid leaving the profession. 

In this study it was found that the participants generally experienced an intention to leave bedside care. This finding is consistent with the findings of other researchers who revealed that the intention to leave is one of the most realistic predictors of real staff turnover^29^. 

It should be noted that employee turnover is not only a financial burden for organizations, but also generates pressures leading to other staff considering leaving clinical care. In addition, because of the loss of manpower and the inability of organizations to rapidly employ replacements there is a subsequent reduction in the quality of the service delivered, with the consequence of reduction in service efficacy and increased patient dissatisfaction^30^.

## Limitations

The experiences of the nurses participating in this study who were working in Medical Science teaching hospitals cannot be extended to nurses who work in different healthcare provision contexts. It is recognized that features of private hospitals are different from public hospitals, therefore more studies are required in order to investigate the experiences of nurses who work in various settings. In addition, human experience is dynamic and the job and work environments of nurses are changed by educational, political, economic and social conditions and expectations of healthcare. It is important therefore to consider the utilization of a longitudinal study to investigate the effects of various changes on the perceptions of nurses regarding their work environment and job. 

## Relevance to clinical practice

The majority of nurses who want to leave the profession are young, of high-quality and in search of new challenges. To keep this category of nurses in the profession, it is necessary to provide new challenges and possibilities for their professional development. This can be accomplished by improving the working conditions and by enhancing the standing of the profession within society, both of which could contribute to higher levels of motivation.

## Conclusion 

The findings of this study provide useful information about the needs of nurses for overcoming intentions to leave bedside care. The identification of this process can help us to recognize emerging problems and offer solutions for them. 
